# Clusters of resilience and vulnerability: executive functioning, coping and mental distress in patients with diffuse low-grade glioma

**DOI:** 10.1007/s11060-024-04704-4

**Published:** 2024-06-19

**Authors:** Floor Gelmers, Marieke E. Timmerman, Femke F. Siebenga, Hiska L. van der Weide, Sandra E. Rakers, Miranda C. A. Kramer, Anouk van der Hoorn, Roelien H. Enting, Ingeborg Bosma, Rob J. M. Groen, Hanne-Rinck Jeltema, Michiel Wagemakers, Jacoba M. Spikman, Anne M. Buunk

**Affiliations:** 1 Department of Clinical Neuropsychology, University of Groningen, University Medical Center Groningen, P.O. Box 30.001, Groningen, AB51, 9700RB The Netherlands; 2 Department of Neurology, University of Groningen, University Medical Center Groningen, Groningen, The Netherlands; 3 Department of Internal Medicine, University of Groningen, University Medical Center Groningen, Groningen, The Netherlands; 4 Department of Psychometrics and Statistics, University of Groningen, Groningen, The Netherlands; 5 Department of Radiation Oncology, University of Groningen, University Medical Center Groningen, Groningen, The Netherlands; 6 Department of Radiology, University of Groningen, University Medical Center Groningen, Groningen, The Netherlands; 7 Department of Neurosurgery, University of Groningen, University Medical Center Groningen, Groningen, The Netherlands; 8 Department of Neurosurgery, Faculty of Medicine Universitas Airlangga, Dr. Soetomo General Academic Hospital, Surabaya, Indonesia

**Keywords:** Low-grade glioma, Executive functioning, Coping, Anxiety, Depression

## Abstract

**Purpose:**

Diffuse low-grade gliomas (dLGG) often have a frontal location, which may negatively affect patients’ executive functions (EF). Being diagnosed with dLGG and having to undergo intensive treatment can be emotionally stressful. The ability to cope with this stress in an adaptive, active and flexible way may be hampered by impaired EF. Consequently, patients may suffer from increased mental distress. The aim of the present study was to explore profiles of EF, coping and mental distress and identify characteristics of each profile.

**Methods:**

151 patients with dLGG were included. Latent profile analysis (LPA) was used to explore profiles. Additional demographical, tumor and radiological characteristics were included.

**Results:**

Four clusters were found: 1) overall good functioning (25% of patients); 2) poor executive functioning, good psychosocial functioning (32%); 3) good executive functioning, poor psychosocial functioning (18%) and; 4) overall poor functioning (25%). Characteristics of the different clusters were lower educational level and more (micro)vascular brain damage (cluster 2), a younger age (cluster 3), and a larger tumor volume (cluster 4). EF was not a distinctive factor for coping, nor was it for mental distress. Maladaptive coping, however, did distinguish clusters with higher mental distress (cluster 3 and 4) from clusters with lower levels of mental distress (cluster 1 and 2).

**Conclusion:**

Four distinctive clusters with different levels of functioning and characteristics were identified. EF impairments did not hinder the use of active coping strategies. Moreover, maladaptive coping, but not EF impairment, was related to increased mental distress in patients with dLGG.

**Supplementary Information:**

The online version contains supplementary material available at 10.1007/s11060-024-04704-4.

## Introduction

Gliomas are the most common primary malignant brain tumors [1, 2]. The slowly growing diffuse isocitrate dehydrogenase (IDH)-mutant astrocytoma and IDH-mutant 1p/19q codeleted oligodendroglioma are generally referred to as diffuse low-grade gliomas (dLGG) [3, 4]. Patients with dLGG have a relatively favorable prognosis [5, 6]. However, dLGG is not curable, so treatment is focused on prolonging overall survival while maintaining quality of life (QoL) [7].

dLGG itself, but also its treatment, can lead to impairments in cognitive functions, such as attention, memory and language, which can affect QoL negatively [8–11]. As most dLGGs are frontally located [12], impairments in executive functions (EF) are common as well [8, 9]. EF refer to a set of higher order cognitive functions including cognitive flexibility, reasoning, planning, and problem solving, which are necessary for goal-oriented behavior and adaptation to novel and complex daily life situations [13].

Adaptation to a changed situation is exactly what is required of patients with dLGG. Most patients are relatively young [2], and fully participating in society, having a family, job and social life. Patients have to cope with symptoms such as cognitive complaints and fatigue [10], while also undergoing intensive treatments. Furthermore, the knowledge of having a life-threatening incurable disease may lead to significant mental distress such as depression and anxiety. Anxiety prevalence rates in patients with dLGG range from 16 to 37% [14, 15] and prevalence rates of depression range from 10–38% [14–17]. Notably, since patients suddenly have to adjust to the consequences of a dLGG and a changed life perspective, the abilities to adequately cope with these changes may be relevant. However, studies investigating which patients with dLGG develop mental distress and why, are lacking.

Coping is defined as “constantly changing cognitive and behavioral efforts to manage specific external and/or internal demands that are appraised as taxing or exceeding the [mental] resources of the person” [18]. Important coping strategies are active coping, passive coping and avoidant coping. Active coping is problem-focused and involves facing challenges and seeking solutions to resolve the stressful situation. Passive coping is characterized by rumination and having a dim view, and avoidant coping involves ignoring and avoiding the situation [19]. People may have a preference but also can use different coping strategies, depending on the situation. Use of passive and avoidant coping strategies is generally linked to depression and anxiety and is therefore considered maladaptive [20, 21]. In contrast, active coping is generally seen as adaptive, but this may differ across situations [18, 20, 21]. To date, only one study investigated effectiveness of different coping strategies in patients with dLGG, finding that avoidant coping was related to a greater degree of mental distress [22].

Active coping involves flexibility, and problem solving, which overlaps with EF abilities. Hence, impaired EF may affect the ability to develop and employ active coping strategies. Consequently, patients may use more maladaptive passive or avoidant coping strategies. This relationship between impaired EF and maladaptive coping has been found in different patient groups, such as patients with multiple sclerosis [23] and stroke survivors [24]. In patients with traumatic brain injury (TBI), some studies find a relationship between EF and coping [25, 26], but not all [27, 28]. Interestingly, a study including both measurements of EF impairments and EF complaints found that EF complaints were more strongly related to passive coping than EF impairments [25]. To date, it has not been investigated in patients with dLGG whether EF impairments and EF complaints are associated with lower use of active problem-focused coping and/or higher use of passive or avoidant strategies, and consequently, increased mental distress.

To gain insight into how EF, coping and mental distress relate to each other in patients with dLGG we aimed to answer the following questions: 1) are there specific patterns of EF, coping and mental distress in patients with dLGG; 2) can we identify demographical, tumor and radiological subgroup characteristics?

## Materials and methods

### Participants and procedure

All adult patients with diffuse IDH-mutated 1p/19q co-deleted oligodendroglioma and diffuse IDH-mutated astrocytoma (WHO grade II or grade III) that were evaluated after surgery and before adjuvant therapy, between November 2017 and April 2023, at the University Medical Center Groningen (UMCG), were eligible for inclusion in this study. In The Netherlands, these patients are preferably treated with proton therapy [29]. All patients receiving proton therapy are consecutively included in a prospective multidisciplinary monitoring program, part of which is a neuropsychological assessment, before start of proton therapy. Exclusion criteria for the present study were no neuropsychological assessment, age younger than 18 years, insufficient proficiency of the Dutch language, previous chemo- or radiotherapy, another neurological disorder, severe psychiatric disorders and alcohol or drug abuse. Data obtainment occurred in compliance with ethical regulations of the Medical Ethical Committee of the UMCG. All participants gave written informed consent at the department of Radiotherapy.

### Neuropsychological assessment

To ensure interpretability of the results, scores are classified based on normative scores as being in the “lowest functioning range” (20% lowest scores of the sample population for questionnaires, percentile scores 20 or lower for EF tests), “moderate functioning range” (20–40% lowest scores of the sample population, percentile score 20–40 for EF tests) or “higher functioning range” (60% highest scores of the sample population or percentile score > 40 for EF tests). See supplementary Table 1 (Online Resource 1) for the corresponding scores on the questionnaires. Raw scores on EF tests are transformed into percentile scores, corrected for age, sex and education, ranging from 1 to 100. A higher score indicates a better performance.

#### Executive function tests

##### Planning

The Zoo Map test, part of the Behavioral Assessment of the Dysexecutive Syndrome (BADS), is used to measure planning ability [30].

##### Cognitive flexibility

The Trail Making Test (TMT) part B is used to measure cognitive flexibility [31].

##### Executive control

The Controlled Word Association Test (COWAT) is used to measure executive control [32].

#### Questionnaires

##### Self-reported EF complaints

The Dysexecutive Questionnaire (DEX) self-rating version is part of the BADS and is used to examine difficulties in everyday situations that involve EF [30]. Scores range from 0 to 80, with a higher score indicating more self-reported EF complaints.

##### Coping

The Utrecht Coping List (UCL) is a self-report questionnaire that examines how participants cope with problems or stressful situations [33]. The items are divided over seven sub-scales from which three scales were used: the active problem-focused coping subscale, the passive coping subscale and the avoidant subscale. Scores range from 0 to 28 (active and passive coping) or 32 (avoidant coping), with a higher score indicating more use of the specific coping strategy.

##### Mental distress

The Hospital Anxiety and Depression Scale (HADS) is a self-report screening scale developed to indicate the possible presence of anxiety and depression in the setting of a medical out-patient clinic [34]. The HADS consists of 14 items and contains two 7-item scales for the separate constructs, both with a score range of 0–21. A higher score corresponds with more symptoms.

### Demographical and Clinical Characteristics

#### Demographics

Age at neuropsychological assessment was categorized as: < 30 years, 30–49 years, 50–59 years and > 60 years.

Educational level was measured on a 7-point scale and based on years of education (YoE): 1 ‘did not finish primary school’ (< 6 YoE), 2 ‘finished primary school’ (6 YoE), 3 ‘unfinished secondary school or special education’ (7–8 YoE), 4 ‘finished secondary school’ (9 YoE), 5 ‘finished vocational training’ (10–11 YoE), 6 ‘finished college education’ (12–16 YoE) and 7 ‘university degree’ (> 16 YoE) [35]. This was subsequently classified as: low (1–4), moderate (5) and high educational level (6–7).

#### Radiological assessment

The radiological appearance of non-tumor involved brain areas was evaluated regarding the amount of white matter T2 hyperintense lesions of a vascular nature by means of the Fazekas score, ranging from 0–3, with 0: non or a single punctate white matter hyperintensity lesion; 1: multiple punctate lesions; 2: beginning confluency of lesions (bridging) and 3: large confluent lesions [36].

#### Tumor assessment

The gross tumor volume (GTV) is based on the pre-radiotherapy MRI scan and defined as the post-surgical tumor bed, including the resection cavity, and the residual T2/FLAIR hyperintense zone.

### Statistical Analysis

Data were analyzed using Statistical Package for the Social Sciences (SPSS) version 28.0 and Latent GOLD version 5.1 [37].

Summary measures were calculated to characterize the dLGG sample, including bivariate correlations between the continuous scores on measures of EF, coping and mental distress.

To identify distinct clusters of patients that show a similar pattern across the domains of mental distress, coping and EF, a latent profile analysis (LPA) was performed. First, gaussian mixture models were estimated based on the 8 outcome parameters (planning, cognitive flexibility, executive control, active coping, passive coping, avoidant coping, anxiety, depression and self-reported EF complaints), varying the number of clusters from 1 up to 10, combined with equal or free within-cluster variances. From these 10 × 2 = 20 models, we selected the model with the lowest Bayesian Information Criterion (BIC) and proper interpretability. The BIC, a statistical index of model fit, is a commonly used index for latent profile models that penalizes the complexity of the model [38].

Subsequently, demographical (age, education, sex), tumor (volume) and radiological (Fazekas score) characteristics of patients were related to the identified clusters. Each characteristic was evaluated as a predictor for cluster membership, using a multinomial logistic regression analysis with adjustment for classification errors [39], and Bonferroni-Holm correction for multiple comparisons (correcting for the five demographic, tumor and radiological characteristics). For all analyses, the nominal significance level was set at 0.05 two-sided.

## Results

151 patients were included for analysis. Sociodemographic and clinical characteristics are shown in Table 1. Neuropsychological characteristics (mean, standard deviation and range) can be found in Online Resource 1, Table 2.

**Table 1:** Sociodemographic and clinical characteristics of patients with dLGG (*n* = 151)

**Characteristic**	
Sex, number of women, *n* (%)	67 (44.4)
Age in years, mean (SD)	42.8 (12.1)
Educational level, mean (SD)	5.2 (1)
Histopathology^*a*^	
Diffuse oligodendroglioma, IDH mutated, 1p/19q co-deleted, *n* (%)	79 (52.3)
Diffuse astrocytoma, IDH mutated, *n* (%)	72 (47.7)
WHO tumor grade^*b*^	
Grade 2, *n* (%)	115 (76.2)
Grade 3, *n* (%)	36 (23.8)
Tumor location^*c*^	
Frontal, *n* (%)	96 (63.6)
Temporal, *n* (%)	21 (13.9)
Insula, *n* (%)	10 (6.6)
Parietal, *n* (%)	21 (13.9)
Occipital, *n* (%)	2 (1.3)
Thalamus/Brainstem, *n* (%)	1 (0.7)
Lateralization^*d*^	
Left-sided, *n* (%)	77 (51.0)
Right-sided, *n* (%)	73 (48.3)
Midline, *n* (%)	1 (0.7)
Time interval between last surgery and NPA in weeks, median (range)	10.6 (4.4—335)
Time interval between last surgery and start RT in weeks, median (range)	10.4 (5.0—335)
Wait and scan policy > 6 months since diagnosis, *n* (%)	51 (33.8)
Type of last surgery	
No craniotomy, only biopsy, *n* (%)	10 (6.6)
Craniotomy under general anesthesia, *n* (%)	85 (56.3)
Advanced craniotomy awake, *n* (%)	56 (37.1)
Extent of tumor resection	
< 25%, *n* (%)	11 (7.3)
25–90%, *n* (%)	96 (63.6)
> 90%, *n* (%)	44 (29.1)
GTV in cc, median (range)	41.1 (1.6—307.0)
Fazekas score	
None or a single punctate WMH lesion, *n* (%)	113 (74.8)
Multiple punctate lesions, *n* (%)	28 (18.5)
Beginning confluency of lesions (bridging), *n* (%)	7 (4.6)
Large confluent lesions, *n* (%)	3 (2.0)
Use of steroids, *n* (%)	2 (1.3)
Active focal epileptic symptoms within 2 weeks before NPA, *n* (%)	14 (9.3)
Use of anti-epileptic treatment	
Mono therapy, *n* (%)	81 (53.6)
Poly therapy, *n* (%)	28 (18.5)
None, *n* (%)	42 (27.7)

dLGG = diffuse low-grade glioma; Educational level = 7-point scale ranging from 1 (no primary school) to 7 (university) according to Verhage (1964); RT = Radiotherapy; IDH = isocitrate dehydrogenase; NPA = Neuropsychological Assessment; GTV = Gross Tumor Volume; WMH = white matter hyperintensities

^*a*^According to the WHO 2021 classification

^*b*^According to the WHO 2016 classification

^*c*^Indicated as the location of the main bulk of tumor

^*d*^Indicated as lateralization of the main bulk of tumor

**Table 2:** Spearman correlation coefficients EF, coping and mental distress

	**1**	**2**	**3**	**4**	**5**	**6**	**7**	**8**
**Executive functioning**								
1. Planning	-							
2. Cognitive flexibility	0.24**	-						
3. Executive Control	0.05	0.37**	-					
4. EF complaints	0.01	0.02	-0.20^a^*	-				
**Mental distress**								
5. Depression	-0.10	0.05	-0.05	0.50**	-			
6. Anxiety	-0.04	-0.09	-0.05	0.48**	0.64**	-		
**Coping**								
7. Active coping	-0.03	0.07	0.12	-0.13^*a*^	-0.17*	-0.12	-	
8. Passive coping	0.06	0.04	-0.06	0.45**	0.48**	0.53**	-0.22**	-
9. Avoidant coping	0.08	0.03	0.08^*a*^	0.43^a^**	0.22**	0.31**	0.00	0.37**

^*a*^Pearson correlation*,* *p < 0.05 (two-tailed), **p < 0.01 (two-tailed)

### Overall correlations between EF, coping and mental distress

EF was not related to indices of coping or mental distress (Table 2). Depression and anxiety were significantly positively but moderately related to self-reported EF complaints, passive coping and avoidant coping. Self-reported EF complaints were also significantly, positively but moderately related to passive and avoidant coping. Depression, but not anxiety, was significantly negatively though weakly related to active coping. Self-reported EF complaints were significantly though weakly correlated with executive control, but not planning or cognitive flexibility.

### Distinct clusters

Out of the 20 LPA models, the model with four clusters and equal within-cluster variance was selected, as it had the lowest BIC and is well-interpretable (Online Resource 1, Table 3). In this model, all clusters differed significantly from each other on means of anxiety, depression, passive coping, active coping, avoidant coping, self-reported EF complaints, mental flexibility and planning abilities, but not on executive control (Table 3).

**Table 3:**
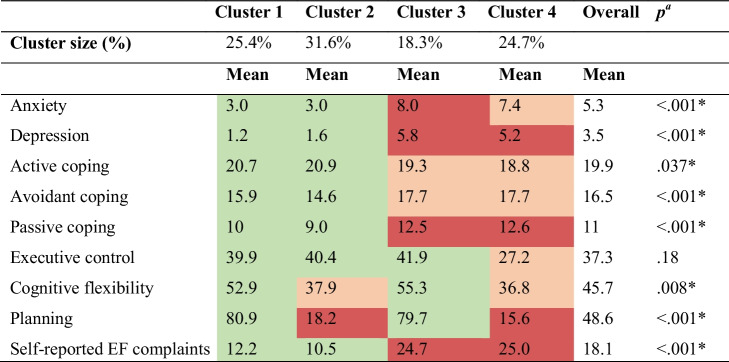
Mean scores of outcome parameters

EF: executive functions; mean EF tests are percentile scores, other scores are raw scores; green = good score, orange = score in the moderate functioning range (i.e. lower than the population mean), red = score in the lowest functioning range (i.e. indicative of possible impairments)

^*a*^Significance level of quality of means across clusters

#### Cluster 1 – Overall good functioning (25.4% of the patients)

Patients within this cluster scored good on EF tests, had few EF complaints (DEX), used adaptive coping strategies (higher use of active and lower use of passive and avoidant coping strategies) and had little mental distress.

#### Cluster 2 – Poor executive functioning, good psychosocial functioning (31.6% of the patients)

Patients within this cluster had few EF complaints, used adequate coping strategies and had little mental distress. However, they scored in the moderate functioning range on cognitive flexibility and in the lowest functioning range on planning.

#### Cluster 3 – Good executive functioning, poor psychosocial functioning (18.3% of the patients)

Patients had elevated mental distress, scoring in the lowest functioning range on depression and anxiety, and used a maladaptive coping strategy (lower use of active and higher use of passive and avoidant coping strategies). In this cluster, scores on EF tests were good, but patients scored in the lowest functioning range on EF complaints.

#### Cluster 4- Overall poor functioning (24.7% of the patients)

Patients within this cluster had high mental distress, scoring in the moderate functioning range on anxiety and in the lowest functioning range on depression. Executive control and cognitive flexibility were in the moderate functioning range, and planning scores and self-reported EF complaints were in the lowest functioning range. Patients in this cluster had a maladaptive coping strategy.

### Demographic and clinical characteristics of the clusters

Across all clusters, significant differences were found in tumor volume (Table 4). Patients in cluster 4 had a significantly larger tumor volume than patients in all other clusters. Cluster 3 consisted of significantly younger patients than cluster 2. Although the other clusters do not significantly differ in age, cluster 3 and 4 consisted of relatively more patients under 50 years old than cluster 1 and 2 (respectively 89.6% and 79% versus 60.1% and 55.9%). Patients in cluster 1 had a higher education level and less (micro)vascular brain damage (lower Fazekas score) compared to cluster 2. No significant differences between clusters were found for sex.

**Table 4:**
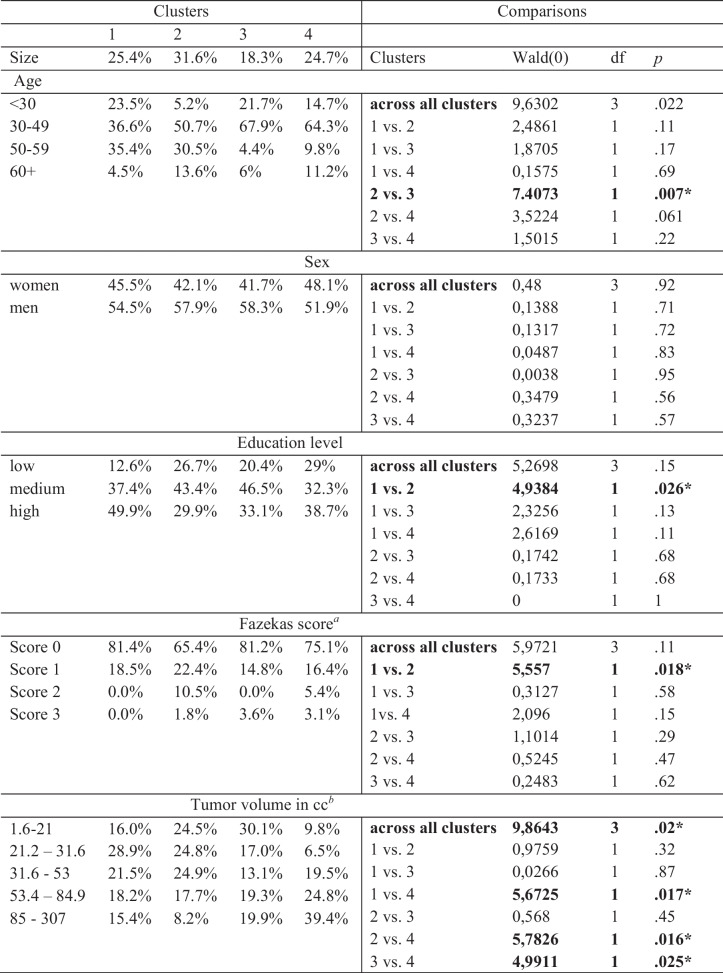
Distribution of demographic and clinical characteristics per cluster

^*a*^Fazekas score: 0: non or a single punctate white matter hyperintensity lesion; 1: multiple punctate lesions; 2: beginning confluency of lesions (bridging) and 3: large confluent lesions [36]

^*b*^Tumor volume is scored on a continuous scale as there is no meaningful categorization known for gross tumor volume

## Discussion

This is the first study in a large group of patients with dLGG investigating patterns of mental distress, coping, EF and self-reported EF complaints, using a Latent Profile Analysis. Four distinct patient clusters were identified. Approximately one quarter of the patients with dLGG had a favorable profile, while all other patients had poorer functioning to at least some extent, with one quarter of patients displaying an overall impaired profile. Furthermore, higher levels of passive and avoidant coping, and lower levels of active coping distinguished clusters with higher levels of mental distress and self-reported EF complaints from those with lower levels of mental distress and EF complaints. However, executive functioning was not a distinctive factor for mental distress, nor for coping. Hence, worse EF does not hamper the use of active coping strategies.

Cluster 1 *‘Overall good functioning’* was a clearly favorable cluster. The three other clusters were less favorable. Cluster 2 *‘Poor executive functioning, good psychosocial functioning’* was characterized by scores in the moderate and lowest functioning range on EF tests. Patients in cluster 2 had a lower educational level and more signs of chronic (micro)vascular brain damage than patients in cluster 1. This is in line with previous research showing a relationship between white matter lesions and executive problems, however this has not been studied specifically in patients with dLGG [40]. Cluster 3 *‘Good executive functioning, poor psychosocial functioning’* consisted of patients with good functioning on EF tests, but scoring in the moderate and lowest functioning range of mental distress, coping and EF complaints. Patients in this cluster were significantly younger than patients in ‘*Overall good functioning’* (Cluster 1). The 4th cluster, *‘Overall poor functioning’,* consisted of patients with low scores on all domains. Notably, patients in this cluster had a larger tumor volume than patients in all other clusters.

Based on the profiles found, it can be tentatively concluded that patients with dLGG in the moderate to lowest functioning range on EF tests are still capable of using active coping strategies, do not resort to maladaptive coping strategies, and do not have elevated levels of mental distress. Furthermore, for patients who use more maladaptive coping strategies, scores on mental distress are higher, which is in line with previous studies [22]. We found that this was irrespective of EF; apparently, coping with emotional situations differs from planning and problem-solving in a structured test setting. Specific matching of stressor and coping strategy has not been investigated in the present study. One could presume that patients do find the situation (dealing with a life-threatening disease) stressful, but it is unknown if other types of stressors need their attention (for example financial difficulties or other (mental) health problems in their family). In individual treatment, this should of course be addressed. On a group level, it can be concluded that passive and avoidant coping are maladaptive in this group, as it is found concurrently with increased mental distress in this study. As the clusters with patients using maladaptive coping strategies consisted of more patients under 50 years old it could be tentatively concluded that younger patients are more prone to using maladaptive coping styles and experiencing mental distress. However, as only cluster 3 differed significantly from cluster 2 regarding age, this has to be interpreted with caution. Previous research into the relationship between age and coping strategies is limited and inconclusive [22, 41].

Patients from the most unfavorable cluster, i.e. having overall impairment, had larger tumor volumes. Previous studies investigating the relation between tumor volume and cognitive functioning are not conclusive [42–44]. Tumor volume in relation to coping or mental distress had not been studied yet in patients with dLGG.

Interestingly, patients with dLGG with maladaptive coping and higher levels of mental distress also report more EF complaints, while not necessarily showing poorer performance on EF tests. This might be due to inaccuracy of patient’s perceptions of their own functioning. Non-cognitive factors such as depression, anxiety and fatigue may lead to a negative bias, causing distorted perception of one’s own (executive) functioning [45–47]. As there were strong correlations between mental distress and self-reported EF complaints, EF complaints seem rather an expression of dissatisfaction than an indication of EF impairments. Therefore, EF complaints may also reflect mental distress in patients with dLGG.

### Limitations

There are a few limitations to this study. First, information about pre-injury patient characteristics on EF, emotional distress and coping styles was not available. Hence, it is not possible to state with absolute certainty that the emotional distress that is found in two of the patient clusters is a result of the dLGG. However, for EF tests we used norm scores corrected for differences in age, sex and educational level, allowing conclusions about impairment. Second, patients were not completely treatment naïve, as they already underwent a biopsy, craniotomy under general anesthesia or advanced awake craniotomy. However, previous studies show that tumor resection does not cause a further deterioration of cognitive functioning in the long-term post-surgery, i.e. most patients return to the pre-surgery level [48, 49]. Third, tumor subtype was not included as tumor characteristic in the analyses. However, no relationship between histopathology (diffuse IDH-mutated astrocytoma versus diffuse IDH-mutated 1p/19q codeleted oligodendroglioma) and cognition was found in two previous studies [12, 49]. Lastly, in general EF and psychosocial functioning in this patient group are relatively good. Therefore, no strong statements can be made about impairments. However, certain subgroups of patients seem to be more at risk for EF impairments or problems in psychosocial functioning, demonstrated by lower scores on these domains.

### Implications

As all domains (mental distress, coping and EF) can be affected in patients with dLGG, individual neuropsychological assessment, including assessment of coping, is of high importance to investigate these aspects in patients with dLGG and give rise to appropriate (neuro) psychological treatment selection and maintain quality of life. As resources for a thorough neuropsychological assessment can be limited, patients with larger tumors are specifically of concern, as they are more at risk for overall worse functioning. Maladaptive coping was found in combination with higher mental distress. Therefore, treatments that decrease passive and avoidant coping should be studied in patients with dLGG. In other neurological patients groups, such as TBI, coping strategy use may be effectively modified through cognitive behavioral therapy (CBT) [50]. Furthermore, CBT as well as acceptance and commitment therapy might be effective in decreasing mental distress in patients with dLGG [51, 52]. As the current study has established the existence of different clusters of patients with dLGG, it would be interesting to investigate if there is a prognostic value of these patient clusters. Preventive and supportive interventions might be timely administered if these clusters are predictive of functioning over time.

### Supplementary Information

Below is the link to the electronic supplementary material.

Supplementary file1 (DOCX 20.6 KB)

## Data Availability

Raw data were generated at the UMCG, subdepartment Neuropsychology. Derived data supporting the findings of this study are available from the corresponding author [FG] on request.
