# Supplementary material for: Clusters of resilience and vulnerability: executive functioning, coping and mental distress in patients with diffuse low-grade glioma
Source: J Neurooncol. 2024 Jun 19;169(1):95–104. doi: 10.1007/s11060-024-04704-4 (PMC11269402; doi:10.1007/s11060-024-04704-4)
Supplement: Supplementary file 1 — Supplementary file1 (DOCX 20.6 KB) [file 11060_2024_4704_MOESM1_ESM.docx]

**Clusters of resilience and vulnerability: executive functioning, coping and mental distress in patients with diffuse low-grade glioma**

Floor Gelmers^1,2,3^, Marieke E. Timmerman^4^, Femke F. Siebenga^1,2^, Hiska L. van der Weide^5^, Sandra E. Rakers^1,2^, Miranda C.A. Kramer^5^, Anouk van der Hoorn^6^, Roelien H. Enting^2^, Ingeborg Bosma^2^, Rob J.M. Groen^7,8^, Hanne-Rinck Jeltema^7^, Michiel Wagemakers^7^, Jacoba M. Spikman^1,2^, Anne M. Buunk^1,7^

^1^ Department of Clinical Neuropsychology, University of Groningen, University Medical Center Groningen, Groningen, The Netherlands

^2^ Department of Neurology, University of Groningen, University Medical Center Groningen, Groningen, The Netherlands

^3^ Department of Internal Medicine, University of Groningen, University Medical Center Groningen, Groningen, The Netherlands

^4^ Department of Psychometrics and Statistics, University of Groningen, Groningen, The Netherlands

^5^ Department of Radiation Oncology, University of Groningen, University Medical Center Groningen, Groningen, The Netherlands

^6^ Department of Radiology, University of Groningen, University Medical Center Groningen, Groningen, The Netherlands

^7^ Department of Neurosurgery, University of Groningen, University Medical Center Groningen, Groningen, The Netherlands

^8^ Department of Neurosurgery, Faculty of Medicine Universitas Airlangga, Dr. Soetomo General Academic Hospital, Surabaya, Indonesia

**Corresponding author**

F.Gelmers, MSc

f.gelmers@umcg.nl

**Table 1 Labels**

|  | **EF tests** | **Active coping** | | **Avoidant coping** | **Passive coping** | **Depression** | **Anxiety** | **EF complaints** |
| --- | --- | --- | --- | --- | --- | --- | --- | --- |
| **Good** | >40 | | ≥ 20 | ≤16 | ≤10 | ≤3 | ≤5 | ≤ 18 |
| **Subclinical** | 20-40 | | 17-19 | 17-19 | 11 | 4 | 6-7 | 19-23 |
| **Clinical** | <20 | | ≤16 | ≥20 | ≥12 | ≥5 | ≥8 | ≥ 24 |

*Note.* Scores on EF tests are percentile scores, scores on other domains are raw scores.

**Table 2 Neuropsychological characteristics**

|  | **M** | **SD** | **Range (min-max)** |
| --- | --- | --- | --- |
| **Executive functioning** |  |  |  |
| 1. Planning | 44,8 | 33,9 | 1-96 |
| 2. Cognitive flexibility | 44,7 | 29,6 | 0-98 |
| 3. Executive Control | 37,2 | 29,8 | 0-99 |
| 4. EF complaints | 17,1 | 9,8 | 0-41 |
| **Mental distress** |  |  |  |
| 5. Depression | 3,1 | 3,1 | 0-17 |
| 6. Anxiety | 5 | 3,5 | 0-18 |
| **Coping** |  |  |  |
| 7. Active coping | 20,0 | 3,5 | 10-28 |
| 8. Passive coping | 10,8 | 2,6 | 7-20 |
| 9. Avoidant coping | 16,3 | 3,2 | 9-26 |

**Table 3 Model fit characteristics**

| **Number of clusters** | **Within-cluster variances** | **LL** | **BIC(LL)** | **AIC(LL)** | **AIC3(LL)** | **Npar** |
| --- | --- | --- | --- | --- | --- | --- |
| 1 | free | -4604,19 | 9298,7 | 9244,389 | 9262,389 | 18 |
| 2 | free | -4472,88 | 9126,38 | 9017,758 | 9053,758 | 36 |
| 3 | free | -4429,62 | 9130,182 | 8967,249 | 9021,249 | 54 |
| 4 | free | -4386,17 | 9133,576 | 8916,332 | 8988,332 | 72 |
| 5 | free | -4356,03 | 9163,608 | 8892,053 | 8982,053 | 90 |
| 6 | free | -4323,4 | 9188,669 | 8862,803 | 8970,803 | 108 |
| 7 | free | -4292,48 | 9217,141 | 8836,963 | 8962,963 | 126 |
| 8 | free | -4269,22 | 9260,927 | 8826,439 | 8970,439 | 144 |
| 9 | free | -4246,58 | 9305,967 | 8817,168 | 8979,168 | 162 |
| 10 | free | -4209,92 | 9322,954 | 8779,844 | 8959,844 | 180 |
| 1 | equal | -4604,19 | 9298,7 | 9244,389 | 9262,389 | 18 |
| 2 | equal | -4512,4 | 9160,275 | 9078,809 | 9105,809 | 27 |
| 3 | equal | -4491,76 | 9164,133 | 9055,511 | 9091,511 | 36 |
| 4 | equal | -4448,31 | **9122,406** | 8986,629 | 9031,629 | 45 |
| 5 | equal | -4434,75 | 9140,426 | 8977,493 | 9031,493 | 54 |
| 6 | equal | -4419,66 | 9155,415 | 8965,326 | 9028,326 | 63 |
| 7 | equal | -4400,29 | 9161,827 | 8944,583 | 9016,583 | 72 |
| 8 | equal | -4381,98 | 9170,364 | 8925,964 | 9006,964 | 81 |
| 9 | equal | -4372,73 | 9197,021 | 8925,465 | 9015,465 | 90 |
| 10 | equal | -4358,6 | 9213,921 | 8915,21 | 9014,21 | 99 |
